# RNA editing in the chloroplast of Asian Palmyra palm (*Borassus flabellifer*)

**DOI:** 10.1590/1678-4685-GMB-2018-0371

**Published:** 2020-01-13

**Authors:** Arpakorn Sakulsathaporn, Passorn Wonnapinij, Anongpat Suttangkakul, Somsak Apisitwanich, Supachai Vuttipongchaikij

**Affiliations:** 1 Center for Agricultural Biotechnology, Kasetsart University, Kamphaeng Saen Campus, Nakhon Pathom 73140, Thailand.; 2 Center of Excellence on Agricultural Biotechnology: (AG-BIO/PERDO-CHE), Bangkok 10900, Thailand.; 3 School of Natural Resource and Environmental Management, Faculty of Applied Science and Engineering, Khon Kaen University, Nong Khai Campus, Nong Khai 43000, Thailand.; 4 Department of Genetics, Faculty of Science, Kasetsart University, 50 Ngarm Wong Wan road, Chatuchak, Bangkok 10900, Thailand.; 5 Center of Advanced studies for Tropical Natural Resources, Kasetsart University, Ngam Wong Wan, Chatuchak, Bangkok 10900, Thailand.; 6 Omics Center for Agriculture, Bioresources, Food and Health, Kasetsart University (OmiKU), 50 Ngarm Wong Wan road, Chatuchak, Bangkok 10900, Thailand.

**Keywords:** Arecaceae, Arecales, chloroplast genome, Commelinids, Phylogeny

## Abstract

We have identified 46 RNA editing sites located in 20 chloroplast (cp) genes of *Borassus flabellifer* (Asian Palmyra palm), family Arecaceae, and tested these genes for supporting phylogenetic study among the commelinids. Among the 46 sites, 43 sites were found to cause amino acid alterations, which were predicted to increase the hydrophobicity and transmembrane regions of the proteins, and one site was to cause a premature stop codon. Analysis of these editing sites with data obtained from seed plants showed that a number of shared-editing sites depend on the evolutionary relationship between plants. We reconstructed a deep phylogenetic relationship among the commelinids using seven RNA edited genes that are orthologous among monocots. This tree could represent the relationship among subfamilies of Arecaceae family, but was insufficient to represent the relationship among the orders of the commelinid. After adding eight gene sequences with high parsimony-informative characters (PICs), the tree topology was improved and could support the topology for the commelinid orders ((Arecales,Dasypogenaceae) (Zingiberales+Commelinales,Poales)). The result provides support for inherent RNA editing along the evolution of seed plants, and we provide an alternative set of loci for the phylogenetic tree reconstruction of Arecaceae’s subfamilies.

## Introduction

RNA editing, a crucial post-transcriptional RNA modification process, allows changes in the genetic information on the primary transcripts in the plastids and mitochondria in many living organisms. Base modifications on the primary transcripts often result in amino acid changes and affect the functionality of the protein products (Tillich *et al.*, 2006). This process has been proposed as an evolutionary mean to restore the original sequence of amino acids of genes that possess mutations (Castandet and Araya, 2011). Generally, 26-64 RNA editing sites are observed in the chloroplast of seed plants (Wakasugi *et al.*, 1996; Huang *et al.*, 2013), and more than a hundred sites have been found in some species such as hornwort (*Anthoceros formosa),* fern (*Adiantum capillus-veneris)* and lycophyte (*Isoetes engelmannii)*. So far, only liverwort (*Marchantia polymorpha*) was found to contain no RNA editing in its plastid genome, and this species is currently used as a baseline for comparing RNA editing in plants (Kugita *et al.*, 2003; Wolf *et al.*, 2004). Besides, RNA editing was found in the plastid of dinoflagellates (Dorrell *et al.*, 2016), but it has not yet been observed in any green microalgae (Chateigner-Boutin and Small, 2011).

Commelinid clade, a group of monocots is categorized by the presence of ferulate/coumarate in the primary cell wall (Chase *et al.*, 1993; Peña *et al.*, 2016). The clade is composed of monophyletic groups of four orders: Arecales (A), Zingiberales (Z), Commelinales (C), Poales (P), and one family named Dasypogonaceae (D). Molecular classification of this clade has long been problematic with conflict topologies. The phylogenetic tree built based on plastome data of 83 taxa showed a monophyletic group of ((P,A)(D,ZC)); however, the relationship within sub-clade of Poales and Arecales, as well as a subclade of (Zingiberales + Commelinales) and Dasypogonaceae, were not strongly supported by bootstrap samplings (Givnish *et al.*, 2010). The topology conflict of the commelinid clade is currently being resolved by dense taxon sampling and availability of plastome sequences. The phylogenetic tree reconstructed from plastome sequences presents that the commelinid clade is comprised of two subclades: a monophyletic clade of Poales being sister to Commelinales+Zingiberales and a monophyletic group of Arecales being sister to Dasypogonaceae ((A,D)(ZC, P)). These subclades are strongly supported by bootstrap analysis (Barrett *et al.*, 2013; Barrett *et al.*, 2016).

*Borassus flabellifer* or Asian Palmyra palm, a member of Arecaceae family, is widespread in Southeast Asia (Pipatchartlearnwong *et al.*, 2017). It is grouped in Coryphoideae subfamily with Phoenix dactylifera (date palm). The economic utilities of *B. flabellifer* are manyfold including building woods, ornamentals, fruits and flower sap for palm sugar production. Currently, only few information is available regarding molecular genetics of *B. flabellifer*. In this work, we sequenced some chloroplast (cp) genes that have been reported to carry RNA editing sites, deduced the pattern of the RNA editing and verified these DNA sequences whether they could support the evolutionary relationship among palm species as well as a deep phylogeny of the commelinids. The results of this study provide insight into the evolution of RNA editing and the evolutionary relationship among subfamilies of Arecaceae family.

## Materials and Methods

### RNA isolation, cDNA synthesis and sequencing

Total RNA of *B. flabellifer* was isolated from unexpanded green fan-shaped leaves using Spectrum^TM^ Plant Total RNA kit (Sigma-Aldrich, USA). cDNA was synthesized using Protoscrip M-MuLV First Strand cDNA kit (NEB, UK) with random hexamer-primers. One microgram of RNA was used for each reaction as recommended in the kit.

Thirty-four Arecaceae cp genes, which were previously reported for carrying RNA editing sites in other plant cp genomes, were chosen for this study (Kugita *et al.*, 2003; Junior *et al.*, 2004; Zeng *et al.*, 2007; Chen *et al.*, 2011; Huang *et al.*, 2013; Uthaipaisanwang *et al.*, 2012). These were *acc*D, *atp*A, *atp*B, *atp*F, *atp*I, *clp*P, *mat*K, *ndh*A, *ndh*B, *ndh*D, *ndh*F, *ndh*G, *ndh*H, *ndh*I, *ndh*K, *pet*A, *pet*B, *psaB*, *psa*J, *psb*C, *psb*H, *rpl*2, *rpl*14, *rpl*20, *rpl*22, *rpl*23, *rpo*A, *rpo*C1, *rps*3, *rps*7, *rps*8, *rps*16, *ycf*2, *ycf*3 and *ycf*4. These 35 transcripts were amplified from the cDNA using PrimeSTAR® GXL DNA Polymerase (Takara-Bio Inc., Japan) with PCR conditions: initial denaturation for 2 min at 98^o^C, followed by 35 cycles of 15 s at 98^o^C, 30 s at 55^o^C or 60^o^C depending on melting temperatures of primers, 60 s at 68^o^C and final extension for 5 min at 68^o^C (see primer sequences in Table S1). PCR products were resolved by 1% agarose gel electrophoresis and purified using a gel extraction kit (Flavorgen, Taiwan) before Sanger’s sequencing (Macrogen, Korea). The transcription sequences were then aligned against predicted RNA editing and cpDNA (GenBank accession number KP_901247; (Sakulsathaporn *et al.*, 2017) using Clustal Omega.

### Analysis of RNA editing sites

The editing sites of the 35 cp genes of *B. flabellifer* were predicted using PREP-Cp (http://prep.unl.edu/cgi-bin/cp-input.pl) with 0.8 cutoff value and PREPACT 3.0 (http://www.prepact.de/prepact-main.php; Lenz *et al.*, 2018). The actual RNA editing sites of these 35 genes were obtained by sequence alignments between the transcripts and the cp DNA sequence (NCBI accession KP_901247) using Clustal Omega.

### Protein structure prediction

Non-synonymous mutation sites and consequent amino acid changes were manually analyzed. The protein secondary structures derived from the edited and non-edited coding sequences were predicted by using SOPMA (http://nhjy.hzau.edu.cn/kech /swxxx/jakj/dianzi/Bioinf7/Expasy/Expasy8.htm). The proportion of alpha helix, extended strand, beta turn and random coil structures between protein derived from edited and non-edited coding sequences were manually counted. The transmembrane structures and signal peptides were predicted by Phobius program (http://phobius.sbc.su.se/instructions.html).

### Comparison of RNA editing sites among plant species

RNA editing sites obtained from *B. flabellifer* (this study), *Cocos nucifera* (identified by Next Generations Sequencing or NGS; Huang *et al.*, 2013) and *Elaeis guineensis* (by NGS; Uthaipaisanwong *et al.*, 2012), in total 53 sites, were compared to those of other 15 plant species. These included six monocots: *S. polyrhiza* (21 sites by NGS), *P. aphrodite* (13 sites by direct cDNA sequencing), *Z. mays* (13 sites by direct cDNA sequencing), *O. sativa* (14 sites by direct cDNA sequencing), *S. officinarum* (12 sites by NGS) and *H. vulgare* (8 sites by direct cDNA sequencing), six dicots: *A. thaliana* (10 sites by direct cDNA sequencing), *P. sativum* (7 sites by direct cDNA sequencing), *A. belladonna* (8 sites by direct cDNA sequencing), *H. niger* (8 sites by NGS), *J. curcas* (7 sites by NGS), *N. tabacum* (10 sites by direct cDNA sequencing) and two gymnosperms: *C. taitungensis* (5 sites by using CURE-Chloroplast software and direct sequencing) and *Pinus thunbergii* (0 sites by direct cDNA sequencing) (Wakasugi *et al.*, 1996; Corneille *et al.*, 2000; Sasaki *et al.*, 2003; Inada *et al.*, 2004; Zeng *et al.*, 2007; Asif *et al.*, 2010; Uthaipaisanwong *et al.*, 2012; Huang *et al.*, 2013; Sanchez-Puerta and Abbona, 2014; Wang *et al.*, 2015; He *et al.*, 2016). The list of genes is presented in Table S2.

### Phylogenetic tree reconstruction

Phylogenetic analyses were carried out using two datasets: (1) DNA sequences of the cp genes carrying RNA editing sites that are shared among monocots and orthologs in *Marchantia* (7 genes: *acc*D, *atp*B, *atp*I, *ndh*B, *ndh*D, *ndh*F and *rps*8) and (2) DNA sequences of the dataset (1), and additional eight cp genes (*mat*K, *rpo*A, *ndh*A, *rpo*C1, *ycf*1, *rpo*C2, *ccs*A and *atp*A) with PIC/length ratio above 0.3. All DNA sequences of cp genes were retrieved from GenBank with accession numbers presented in Table S3. Multiple sequence alignment was performed for each gene using MACSE (Ranwez *et al.*, 2011), and aligned sequences were concatenated using SequenceMatrix (Vaidya *et al.*, 2011). The phylogenetic trees were reconstructed based on maximum likelihood (ML) and Bayesian (Bayes) methods with GTR+I+G model using RAxMLGUI (Silvestro and Michalak, 2012) and MrBayes (Ronquist *et al.*, 2012), respectively. This evolutionary model was determined by jModelTest based on AICc value (Darriba *et al.*, 2012). Ten-thousand replicates and 10M generations with stop value at 0.01 were applied to calculate statistical supports for maximum likelihood and Bayesian phylogeny, respectively. *Doryanthes palmeri* (Asparagales) was used as an outgroup.

## Results

### RNA editing sites in the cp genome of *B. flabellifer*

**Figure 1:**
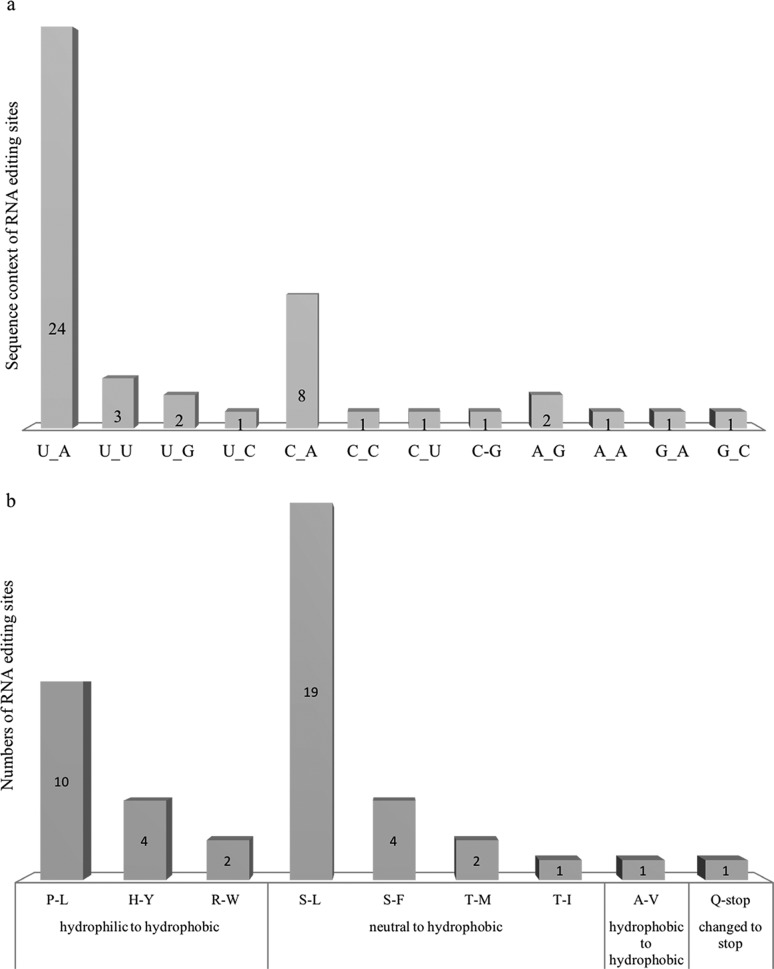
The frequency of sequence contexts of RNA editing sites (a) and amino acid changes caused by RNA editing (b) in the chloroplast of *B. flabellifer.*

Based on previous reports on RNA editing in the transcripts of 35 chloroplast genes in monocots, we aimed to analyze the transcripts of these genes in the cp genome of *B. flabellifer.* PREP-Cp program predicted 67 potential editing sites located in 19 out of the 35 target genes, while PREPACT 3.0 predicted 57 editing sites in 23 genes. We sequenced the transcripts of these 35 genes, aligned them against the *B. flabellifer* cp DNA (NCBI accession KP_901247) and found that there were in total 46 RNA editing sites located in 20 genes (Table S4). Although only 32 sites out of 67 predicted sites were correct (47.76%), it is important to note that the PREP-Cp was unable to predict editing sites at the third base of the codon. The numbers of the observed editing sites per gene were varied from one to several, and most frequently edited genes were *ndhB*, *ndh*D and *rpo*C1, with 11, 4 and 4 editing sites, respectively. All of the 46 editing sites were with C-to-U editing type. Examination of the adjacent sequences of (_C_) editing sites revealed that the frequency of nucleotides preceding the C editing sites were U (65.21%), C (23.91%), A (6.52%) and G (4.34%), and that of nucleotides following the editing sites were A (73.91%), G (10.67%), U (8.70%) and C (5.52%). Thus, U_A is the highest context of RNA editing sites (52.17%) (Figure 1a).

Analysis of the editing sites based on base positions within codons showed that seven (15.21%), 37 (80.43%) and two (4.36%) editing sites were located at the first, second and third base, respectively. Follow-up analysis of the edited coding sequences showed that 43 out of 46 editing sites caused amino acid changes, one site generated a premature stop codon and the other two were silent mutations. The amino acid changes were found to be preferably converting neutral (26 sites) and hydrophilic (16 sites) amino acids to hydrophobic amino acids (Figure 1b). The most frequent amino acid alteration was from serine to leucine, the neutral to the hydrophobic amino acid, followed by alteration from proline to leucine, the hydrophilic to hydrophobic amino acid.

### Impacts of RNA editing on polypeptides and protein structures

**Figure 2:**
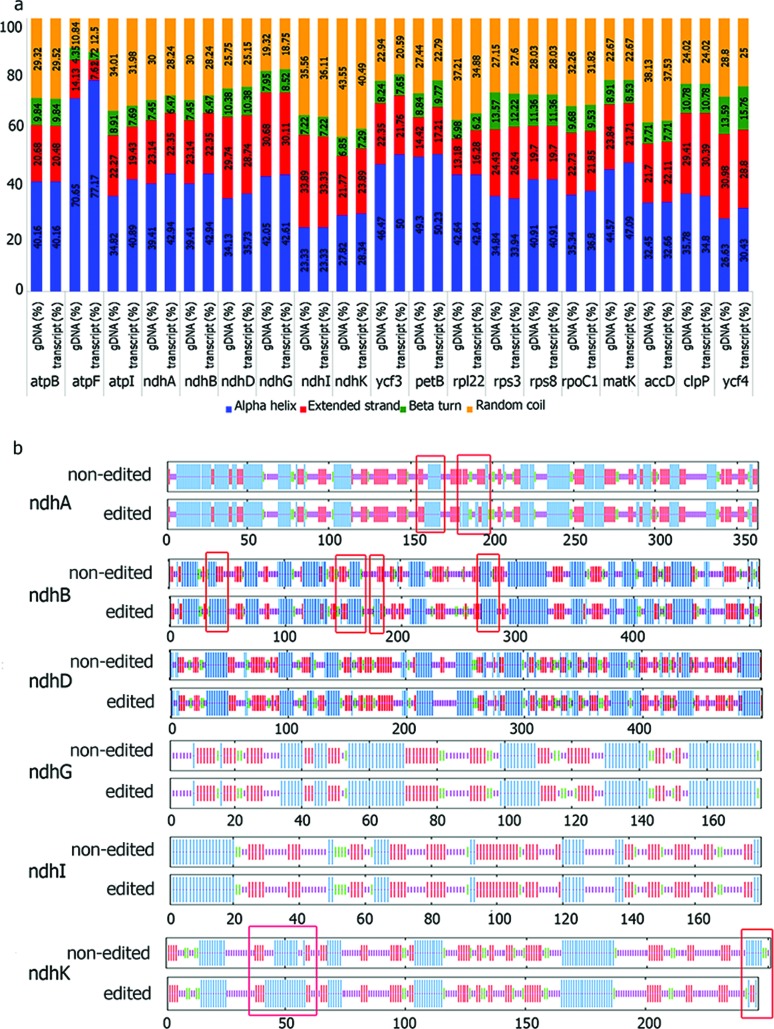
Comparison of protein structure before and after editing. (a) The proportion of protein secondary structures (in percentages) derived from gDNA sequence and transcript sequences. Blue: alpha-helix, red: extended strand, green: beta-turn, orange: random coil. (b) Patterns of transmembrane regions in NDH proteins compared between non-edited and edited sequences. Squares indicate altered transmembrane regions.

To evaluate the impact of the RNA editing in *B. flabellifer* on the protein structures, we analyzed the modified protein sequences using SOPMA program. Analysis of the predicted secondary structures showed that the amino acid changes could result in the increase of the alpha helix structure, extended strand and beta-turn or random coil (Figure 2a). In particular, the RNA editing of *ndh*A, *ndh*B, *ndh*D, *ndh*F, *ndh*G, *ndh*I and *ndh*K genes showed increases in the alpha helix of their encoded protein: NADH-plastoquinone oxidoreductase. As the observed RNA editing mainly resulted in hydrophobic amino acid and changes in the hydrophobicity have been suggested to affect the transmembrane properties of proteins (Kugita *et al.*, 2003; Wang *et al.*, 2015; He *et al.*, 2016), we compared the predicted transmembrane and signal peptide regions between the edited and non-edited versions of NdhA, NdhB, NdhD, NdhF, NdhG, NdhI and NdhK proteins. While there was no alteration of the signal peptide regions, expansions of transmembrane regions were observed in many regions of these proteins, particularly NdhA, NdhB, and NdhK (Figure 2b). Especially, amino acid changes at ndhA_S159L and ndhA_S189L were found to generate two new transmembrane regions between the amino acid positions 159 and 169 and the positions 182 and 208, respectively*.* Moreover, NdhB and NdhK proteins resulted from RNA editing including *ndh*B_50SL*, ndh*B_156PL*, ndh*B_181TM, *ndh*B_277SL and *ndh*K_44SL gained new transmembrane regions between codon 35 and 50, 155 and 181, 178 and 182, 270 and 283, and 43 and 51, respectively.

### RNA editing sites among *B. flabellifer* and other plant species

**Figure 3:**
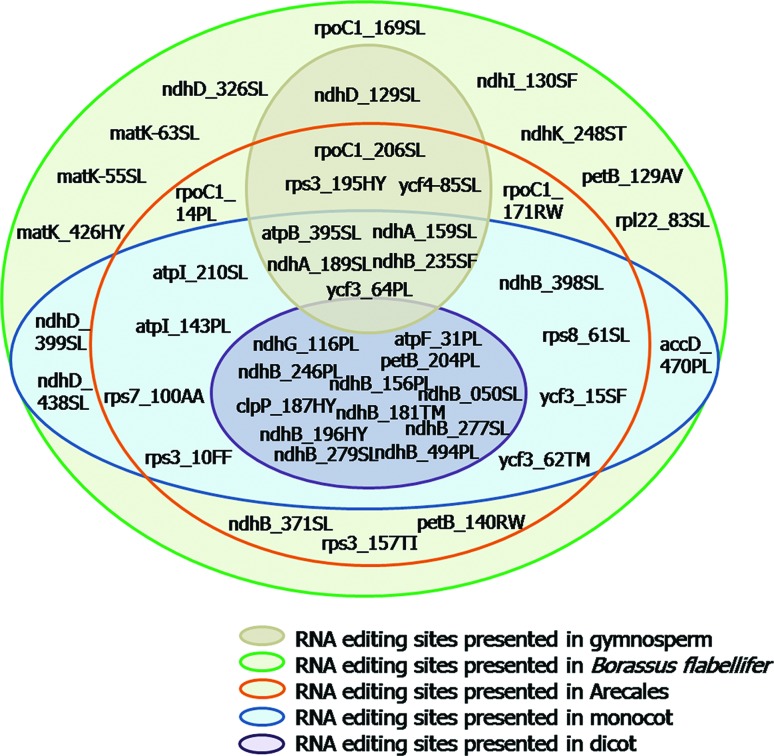
Comparisons of 46 RNA editing sites observed in *B. flabellifer* to those observed in other 16 plants, which include gymnosperms, dicots, monocots and palms.

The RNA editing sites and resulted amino acid alterations observed in *B. flabellifer* were compared to those previously observed in other 17 plant species that belong to the order Arecales (palms), monocots, dicots, and gymnosperms (Figure 3 and Table S2). Among 46 editing sites observed in *B. flabellifer*, we found that nine sites (*ndh*D_326SL, *ndh*I_130SF, *ndh*K_248QST, *pet*B_129AV, *rpl22*_83SL, *rpo*C1_169SL, *mat*K-55SL, *mat*K-63SL and *mat*K426HY) were unique to this species, and 33 and 28 sites were shared among Arecales and monocots, respectively. Furthermore, 12 sites were shared between monocots and dicots, and five sites were shared between monocots and gymnosperms. This result suggested that more common RNA editing sites are likely to be found in evolutionary related species and that these editing sites could be useful for studying the evolutionary relationship of closely related species.

### Phylogenetic trees of commelinids based on cp genes carrying RNA editing sites

**Figure 4:**
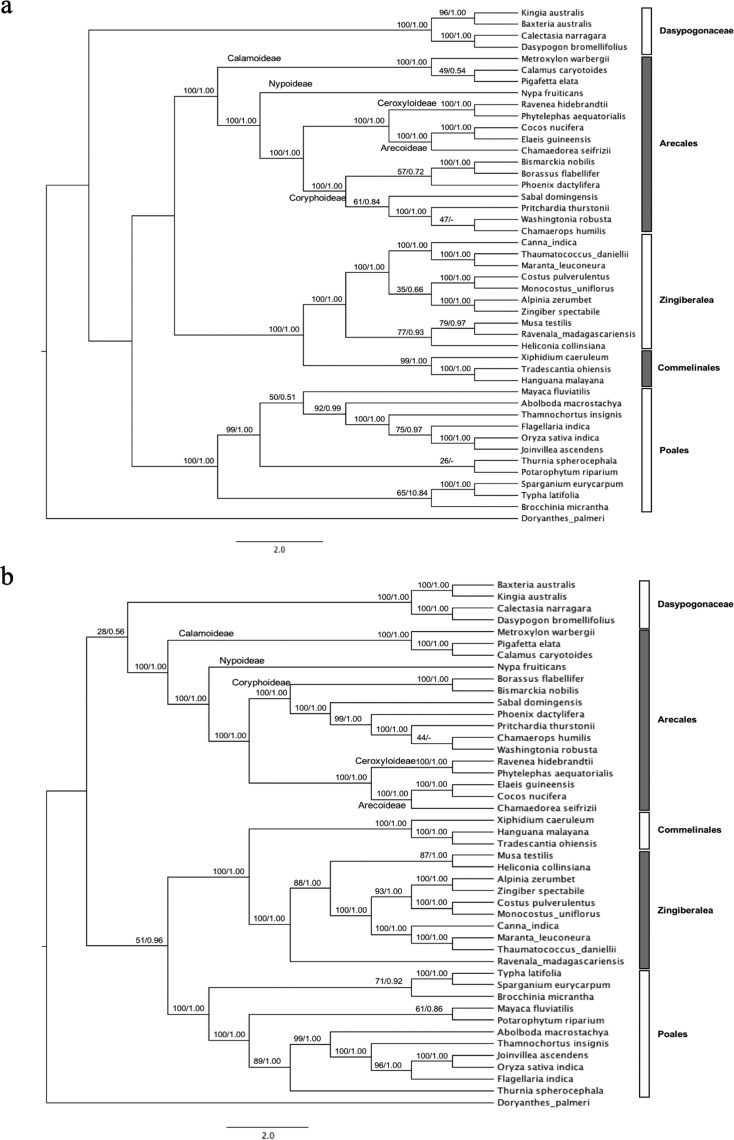
Cladograms of commelinids reconstructed from seven cp genes (a) and 15 cp genes (b) based on maximum likelihood and Bayesian method with GTR+I+G model. The numbers on each branch present the bootstrap support (BP) and posterior probability value (PP), respectively. 10,000 replicates and 10M generations with stop value at 0.01 were applied to calculate statistical supports for maximum likelihood and Bayesian phylogeny, respectively.

Following the orthologous genes with common RNA editing sites identified among the commelinid clade, we tested whether these genes are able to support the evolutionary relationship among the orders within this clade. First, we used the DNA sequence of seven RNA edited genes; *acc*D, *atp*B, *atp*I, *ndh*B, *ndh*D, *ndh*F and *rps*8, which have orthologous editing sites among the monocots and are orthologues to *M. polymorpha.* The evolutionary relationship among the orders was reconstructed as (D(P(A,ZC))) (Figure 4a). This tree topology did not correspond well with the report from Barratt *et al.* (2016), which used an extensive dataset including the complete coding sequence of 75 genes of the cp genomes of 132 monocot taxa. This result indicated that a dataset using these seven gene sequences is insufficient for reconstructing a representative phylogeny for the commelinid clade members. We, therefore, built another phylogenetic tree using a new dataset, which included the seven RNA edited cp genes, additional four RNA edited cp genes: *mat*K, *rpo*A, *ndh*A and *rpo*C1, and four non-RNA edited cp genes: *ycf*1, *rpo*C2, *ccs*A and *atp*A (Figure 4b). These additional eight genes were selected based on the number of parsimony-informative characters (PICs), which the PIC/length ratios of these genes were above 0.3 (Barratt *et al.*, 2013). Based on these 15 cp genes, the reconstructed phylogeny showed ((D,A)(P,ZC)) clustering, which was similar to that proposed by Barrett *et al.* (2016). Because *ycf1* is often considered variable, we therefore tested the phylogenetic analysis using 14 cp genes (without *ycf1*) and found that the clustering pattern remained the same with less bootstrap support than that of 15 cp genes (Figure S1). Thus, this result showed that the 15 cp sequences were able to provide reliable reconstruction of the topology of the commelinid clade.

## Discussion

In this work, we reported for the first time RNA editing in the chloroplast of *B. flabellifer*. Analysis of 35 cp genes revealed authentically 46 RNA editing sites in 20 genes, and the editing was all with C-to-U type. U_A is the highest editing context observed in this species. Most of the editing occurred at the first and second base of codons resulting in amino acid changes in 43 out of 46 codons. The codon changes resulted in increases of the hydrophobicity and extension of the transmembrane regions in a number of proteins, particularly NdhA, NdhB, NdhD, NdhG, NdhI, and NdhK. Comparison of the amino acid changes via RNA editing from 18 plant species showed high numbers of shared editing sites in closely related species suggesting the use of DNA sequences of the genes carrying these editing sites for evaluating the evolutionary relationship. The phylogenetic tree built based on DNA sequences of seven orthologous genes carrying RNA editing sites could not represent the deep phylogeny of commelinids; however, this tree could well represent the evolutionary relationship among subfamilies of Arecaceae, suggesting that these seven loci could potentially be used for solving the evolutionary relationship among the members of the family Arecaceae.

All of the 46 RNA editing sites observed in the chloroplast of *B. flabellifer* demonstrated only the C-to-U type. Previous reports show that the C-to-U type is widespread among seed plants, whereas the reverse editing U-to-C type is present in some lower land plants including a hornwort (*Anthoceros formosae*)*,* a fern (*Adiantum capillis-vneris*) and a lycophyte (*Selaginella uncinata*) (Yoshinaga *et al.*, 1996; Kugita *et al.*, 2003; Wolf *et al.*, 2004; Chateigner-Boutin and Small, 2010; Grewe *et al.*, 2011), but not in liverworts, mosses and gymnosperm (Rüdinger *et al.*, 2009; Wu *et al.*, 2014; Guo *et al.*, 2015). This observation suggests that the U-to-C type was likely to be originated in the common ancestor of hornwort and tracheophytes and was lost via the separation between mosses and hornwort as well as via the separation between ferns and seed plants (Chateigner-Boutin and Small, 2010). However, in subfamily Arecoideae, Uthaipaisanwong *et al.* (2012) showed that both editing types are present in *Elaeis guineensis* (oil palm), while *Cocos nucifera* (coconut) was reported to possess only C-to-U type (Huang *et al.*, 2013). This contradiction suggests that the U-to-C type may not be completely lost during the separation between ferns and seed plants or it may arise during the evolution of seed plants. Since we were unable to analyze all of the cp transcripts, we could not rule out the possibility for the U-to-C type in *B. flabellifer.* Further study to identify the U-to-C editing in *B. flabellifer* is needed for understanding the evolution of RNA editing in the cp genome in the family Arecaceae.

The predominant U_A context of RNA editing sites in *B. flabellifer* is similar to the context bias in other seed plants including *Phalaenopsis aphrodite, Nicotiana tabacum, Zea mays, Pinus thunbergii, Atropa belladonna*, and *Arabidopsis thaliana* (Tillich *et al.*, 2006; Zeng *et al.*, 2007). It has been proposed that approximately 30 nucleotide sequences surrounding the editing site are the recognized region of RNA editing factors, and conserved sequences within this region have not yet been identified (Okuda and Shikanai, 2012). Likewise, no conserved sequence could be deduced from alignments of 30 nucleotide-regions surrounding the 46 editing sites observed in the cp genome of *B. flabellifer*.

The increases in the hydrophobicity of the chloroplast proteins through the majority of the RNA editing in *B. flabellifer* are a widespread phenomenon. Indeed, our analysis showed evidence of the impacts of this hydrophobicity on the protein secondary structures. It was proposed that these consequently cause the extensions of transmembrane regions in many chloroplast proteins, particularly Ndhs, which increase the stability of the NDH complex and makes it tightly bind to the thylakoid membrane (Jobson and Qiu, 2008).

Generally, RNA editing can generate both new start and stop codons. Many cp genes in angiosperm gain new start codons through RNA editing, which modifies C-to-U of ACG initiator codon to AUG standard start codon such as that in *ndhD*, *psbL* and *rpl2* (Sugiura *et al.*, 1998; Wu *et al.*, 2014). However, the analysis of RNA editing in *B. flabellifer* demonstrated the loss of ACG RNA editing in the initiator codon in *ndh*D and *rpl*2. The premature stop codon observed in the *ndh*K resulting in deleted eight amino acids at the C-terminus of NdhK protein has never been observed in the chloroplast of plants in the family Arecaceae, though this was previously observed in other monocots such as *Wolffia australiana* (NC_015899.1), *Colocasia esculenta* (NC_016753.1) and *Zea mays* (NC_001666.2). Although we have analyzed 35 cp genes of *B. flabellifer* for RNA editing, in which 20 genes are positive, the assessment of RNA editing in this species is still far from being complete. There are other 45 out of 79 protein coding genes in the *B. flabellifer* cp genome left to be analyzed (Sakulsathaporn *et al.*, 2017).

The evolutionary relationship among the orders of commelinid was initially reconstructed using several sets of organelle genes including a set of cp genes: *rbc*L, *atp*A, *mat*K and *ndh*F and a set of chloroplast and mitochondrial genes: *rbc*L, *mat*K, *atp*1, and *nad*5. Several tree topologies were proposed such as ((ZC,A)(D,P)), (A(ZC(D,P))), (A(D(P,ZC))), (D(A(P,ZC))), ((P,ZC)(A,D)) and (A(P(ZC,D))) (Kress *et al.*, 2002; Davis *et al.*, 2004; Chase *et al.*, 2006; Graham *et al.*, 2006; Specht *et al.*, 2006; Saarela *et al.*, 2008; Givnish *et al.*, 2010). Barrett *et al.* (2013) reported that both the single most parsimonious tree and the best-scoring likelihood tree reconstructed based on plastome sequences of 46 taxa supported the ((A,D)(ZC,P)) topology. Nonetheless, Barrett *et al.* (2013) also proposed six alternative topologies: (ZC(A(D,P))), ((ZC,A)(D,P)), (A(ZC,(D,P))), (A(D(P,ZC))), (D(A(P,ZC))), ((P,A)(ZC,D)), which were not significantly less likely present the evolutionary relationship among orders belonging to the commelinid clade. From our observation that the closely related plants have more shared editing sites compared to the distantly related plants, the phylogenetic tree reconstructed based on seven cp genes with RNA editing sites presented (D(P(A,ZC))) topology, which was reported as significantly less likely topology compared to the best-scoring likelihood tree (Barrett *et al.*, 2013). These results suggested that only the presence of RNA editing sites would not be a good criterion for selecting orthologous genes to represent deep phylogeny, the evolutionary relationship among plant orders.[Bibr B17]

Most recently, Barrett *et al.* (2016) reported a maximum likelihood phylogenetic tree with ((A,D)(ZC,P)) topology, which was reconstructed from plastome sequences comprised of 75 protein-coding genes. The (A,D) and (ZC,P) clusters of the trees reconstructed from 75 protein-coding genes were supported by 90% and 96% bootstrap samples, respectively, while those that reconstructed from 46 protein-coding genes previously reported by Barratt *et al.* (2013) were supported by 72% and 85% bootstrap samples, respectively. Compared to our topology as shown in Figure 4b, the tree was reconstructed using only 15 protein-coding genes from the cp genomes of plants belonging to the family Arecaceae, and this can provide a similar topology to those that used large datasets, though the (A,D) and (ZC,P) clusters were supported by only 28% and 51% of bootstrap samples, respectively. Noting that these 15 protein-coding genes comprised of seven edited genes, four edited genes with high PICs and four non-edited genes with high PICs. The similarity of the overall topology suggested that adding DNA sequence data of the genes carrying a high number of PICs could improve the tree topology presenting evolutionary relationship among orders of the commelinids. This result also supports Barratt *et al.* (2013) that only a particular set of cp genes with high PICs could well represent the deep phylogeny of the commelinids.

It was notable that even though the two phylogenetic trees reconstructed using seven and 15 protein-coding cp genes present different evolutionary relationship among several orders of the commelinid clade, the close relationship between Zingiberales (Z) and Commelinales (C) is maintained as supported by 100% of bootstrap samples. And, each order presents a monophyletic cluster of plant species supported by at least 99% bootstrap samples. By focusing only on the evolutionary relationship among the subfamilies of palms (family Arecaceae, Order Arecales), both phylogenetic trees support that the relationship among these subfamilies as (Calamoideae, (Nypa, (Coryphoideae (Ceroxyloideae, Arecoideae)))) with almost 100% bootstrap samples. This relationship corresponds well with the clusters that were previously reconstructed by using nine plastid markers, four nuclear markers, a morphological dataset and a RFLP dataset (Baker *et al.*, 2011; Faurby *et al.*, 2016) as well as the phylogenetic relationship reconstructed by using 75 protein-coding genes of 132 taxa with 100% of bootstrap (Barratt *et al.*, 2016). Hence, the use of the seven genes, which have RNA editing and share with monocots, is sufficient for verifying the evolutionary relationship among the subfamilies of the Arecaceae family.

## Supplementary material

The following online material is available for this article:

Figure S1 Cladograms of commelinids reconstructed from 14 cp genes (without *ycf1*) based on maximum likelihood and Bayesian method with GTR+I+G model.

Table S1 Oligonucleotide primers for amplification of 35 cp genes.

Table S2 Comparison of RNA editing sites in chloroplast transcripts from 18 plant species.

Table S3 Accessions numbers of cp genomes from 59 plant species.

Table S4 Predicted and experimentally identified RNA editing sites in 35 chloroplast genes of *B. flabellifer.* + = editing site, - = no editing site and # = not predicted by the PREP-Cp.
