# Supplementary material for: RNA editing in the chloroplast of Asian Palmyra palm (Borassus flabellifer)
Source: Genet Mol Biol. 2020 Jan 13;42(4):e20180371. doi: 10.1590/1678-4685-GMB-2018-0371 (PMC7206934; doi:10.1590/1678-4685-GMB-2018-0371)
Supplement: Supplementary file 1 [file 1415-4757-GMB-42-4-e20180371-suppl2.pdf]

Supplementary Material to: "RNA editing in the chloroplast of Asian Palmyra palm (*Borassus flabellifer*)"

Table S1 - Oligonucleotide primers for amplification of 35 cp genes.

Primers	Sequence 5'-3'
accD-F	TGTTACGTTATTGTCATCTTATTAGTT
accD-R	ATTACCCGAAACTTCTAATTCTTAT
atpA-F	TGTACTAAGAATAAGAGGGAGAAGTTG
atpA-R	CTAATGGTAACCCTTCGAGCC
atpB-F	CAGTACACAAAGATTTAAGGTCATT
atpB-R	TATGAGAATCAATCCTACCCCT
atpF-F	AAGTAAAAGTAAAAGGGCTAATCTGT
atpF-R	TTAGTCCTATCTATAAAAGGAGAGCAT
atpI-F	CCACAATATCGTCTATCTTTTTTCCTA
atpI-R	GTTATGATGTTATGTAATTTCTTGGTATCG
clpP-F	CGAATTATGGTTTGAAATGGA
clpP-R	TAATGCCCATTGGTGTCCC
matK-F	TCTATTCATAAGTCTCATCACGTCA
matK-R	TTCTAAATATCCTTGTAATTCTTCCAT
ndhA-F	ATAGTGAAACAAGTTGGGAAGAAG
ndhA-R	GAGAAGTTGATCGTTGAAATGATAA
ndhB-F	TAAAAGAGGGTATCCTGAGCAAT
ndhB-R	TTGGATGCAGTTACTAATTCA
ndhD-F1	GAGCACGGGCTTTTCTGGTC
ndhD-R1	CGAATCAATCCATATGCTCCC
ndhD-F2	CCCTACATACATGGTTACC
ndhD-R2	TAATGAAACACTATCTATAAAAGTA
ndhF-F1	GAAAGAGATAAGAATTGGTGAATC
ndhF-R1	AATACCACAAAGAGAAAGTGTACC
ndhF-F2	CTTATCGAGCGGCTTTATTC
ndhF-R2	GCTGTACGAAATGAAATGTCAC

Primers	Sequence 5’-3’
ndhG-F	TCTAAGTATTTATTATTGACGAGCCAT
ndhG-R	TTTAATAATGGATTTACCTGGACC
ndhH-F	TCTGTCGTATCAATTATCATTTCAAC
ndhH-R	AAGGAAGAAAAAATATGACTATACCG
ndhI-F	ATCTTAGTAATTGGTAATCGTTCTTGA
ndhI-R	CAATATGTTCTCTATGGTGACCG
ndhK-F	CGCCGTTTCTTATTCTGCAC
ndhK-R	AGGAGCATTGGAATGGTCTTAAC
petA-F	CGGGATCAATGATTGGACA
petA-R	ATGTTAAGGAATTTCTGGACCTAG
petB-F	GAAAGTCCCAGTTTTTCAATCA
petB-R	TGTAGCAATGAATCCATATTTGTA
psaB-F	GGACGTGCTGTAGGAGTAACCCAT
psaB-R	CAAGAAGGCAAATCCTCCCTCTC
psaJ-F	GGACAGGAAAATCTCATCTATTG
psaJ-R	TCCCCAAAATTCGATCTTAG
psbC-F	CTCTGAACCTCCGTGCCTATGAC
psbC-R	GAATCCCTCTCTCTCCTTTTGTTTGT
psbH-F	GAGATGGCGACTAGGGTTGCTG
psbH-R	CAAATAAATCTCACGGTCGAACTACC
rpl2-F	TAATGGTTTGGGTCGTGTATC
rpl2-R	CAATATCCCCAGTACTTTTTTTAT
rpl14-F	ATAGTGTCCCTACCCATAACGAAC
rpl14-R	GAACTAAACAAAAGGGATATTGAGG
rpl20-F	GTTCGTTCATTCCTACTACTTATC
rpl20-R	TCATAGTAGCAAAAGCCATTG
rpl22-F	TGATTTAGATTTTTCCTTTAATAC
rpl22-R	GGGATGACCTTATGATAAAGAAC
rpl23-F	GTATCGCCGTGTTATTAAGTATTTTG
rpl23-R	CTGGAACTCATAGGGAAGAAAATG
rpoA-F	GATCCATTCCGGGAAAATACG
rpoA-R	GTACGTGATGTAACACCTATGCCA

Primers	Sequence 5’-3’
rpoC1-F	CGACAATGGGCCTGAGTCAG
rpoC1-R	AACCCCGAGGATGCCATC
rps3-F	TCTCGTTTACAAATATCCAAACTCGT
rps3-R	TGAATGGAAAGAGAACATAATAGAAAAAT
rps7-F	ATGGATTAACGAAAATGTGCAAAA
rps7-R	TGATTCTTGAACCTCTTTCACGC
rps8-F	GCAAAATACTTAATAACACGGCGATAC
rps8-R	TCGTTATGGGTAGGGACACTATTGCC
rps16-F	AGTTCTTTCTTAAATAATTCTGCCTTC
rps16-R	GTTTGAAATAAAATTAGAGGAATGTTATGG
ycf2-F1	GAGCTAAGGTCCAAAATATGGAATAAAC
ycf2-R1	GTCAAATACCTAACGACAAACTCCTATG
ycf2-F2	AATCGGTCTATTTTCGGCGTC
ycf2-R2	TTCCTCAATATCTTGTTCATTCGC
ycf2-F3	TGTTAGAGGTGTTGCAGAAATGTC
ycf2-R3	CGATCAAACAGAGATTCAGCAAC
ycf2-F4	GGATGAATTGAGACGGTATTTTG
ycf2-R4	CGCTTTAACCATTCAGCCATG
ycf3-F	AGGAAGGGGTATAATGAAATTCTT
ycf3-R	AATTTGAGCGGAAGGTCCAC
ycf4-F	CAATCGAATGCAACTGGATCTAGT
ycf4-R	TTTCTTCAGTTCATTTCAAAATACTTCA
