# Supplementary material for: RNA editing in the chloroplast of Asian Palmyra palm (Borassus flabellifer)
Source: Genet Mol Biol. 2020 Jan 13;42(4):e20180371. doi: 10.1590/1678-4685-GMB-2018-0371 (PMC7206934; doi:10.1590/1678-4685-GMB-2018-0371)
Supplement: Supplementary file 2 [file 1415-4757-GMB-42-4-e20180371-suppl3.pdf]

# Supplementary Material to: “RNA editing in the chloroplast of Asian Palmyra palm (*Borassus flabellifer*)”

Table S2 - Comparison of RNA editing sites in chloroplast transcripts from 18 plant species.

| Gene | *Ginkgo biloba* | *Cycas taitungensis* | *Pinus thunbergii* | *Borassus flabellifer* | *Cocos nucifera* | *Elaeis guineensis* | *Hordeum vulgare* | *Oryza sativa* | *Phalaenopsis aphrodite* | *Saccharum officinarum* | *Spirodela polyrhiza* | *Zea mays* | *Arabidopsis thaliana* | *Atropa belladonna* | *Hyoscyamus niger* | *Jatropha curcas* | *Nicotiana tabacum* | *Pisum sativum* |
|---|---|---|---|---|---|---|---|---|---|---|---|---|---|---|---|---|---|---|
| accD_470PL | - | - | - | + | - | - | # | ^ | + | ^ | - | ^ | - | # | # | - | - | - |
| atpB_395SL | + | - | - | + | + | + | - | - | + | - | - | - | - | - | - | # | - | - |
| atpF_31PL | - | - | - | + | + | + | # | - | + | - | + | - | + | + | + | + | + | - |
| atpI_143PL | - | - | - | + | + | - | # | - | + | - | - | - | - | # | # | # | - | - |
| atpI_210SL | - | - | - | + | + | - | # | - | + | - | - | - | - | # | # | # | - | - |
| clpP_187HY | - | - | - | + | + | - | # | - | + | - | - | - | + | # | # | + | - | - |
| matK_55SL | - | - | - | + | - | - | # | - | - | - | - | - | - | # | # | # | - | - |
| matK_63SL | - | - | - | + | - | - | # | - | - | - | - | - | - | # | # | # | - | - |
| matK_426HY | - | - | - | + | - | - | # | - | - | - | - | - | - | # | # | # | - | - |
| ndhA_159SL | + | + | ^ | + | + | - | # | + | ^ | + | + | + | - | - | - | # | - | - |
| ndhA_189SL | - | + | ^ | + | + | - | + | + | ^ | + | - | + | - | - | - | # | - | - |
| ndhB_50SL | - | - | ^ | + | + | - | + | - | ^ | - | - | - | + | - | - | # | + | + |
| ndhB_156PL | - | - | ^ | + | + | - | + | + | ^ | + | + | + | + | + | + | # | + | - |
| ndhB_181TM | - | - | ^ | + | + | - | - | - | ^ | - | + | - | - | + | + | + | - | - |
| ndhB_196HY | - | - | ^ | + | + | - | - | + | ^ | + | + | + | + | - | - | + | + | + |
| ndhB_235SF | + | + | ^ | + | + | - | - | + | ^ | - | + | - | - | - | - | # | - | - |
| ndhB_246PL | - | - | ^ | + | + | + | + | + | ^ | + | + | + | - | + | + | + | + | + |
| ndhB_277SL | - | - | ^ | + | + | - | + | + | ^ | + | + | + | + | + | + | + | + | + |
| ndhB_279SL | - | - | ^ | + | + | - | + | + | ^ | - | + | - | + | + | + | + | + | + |
| ndhB_371SL | - | - | ^ | + | + | - | - | - | ^ | - | - | - | - | - | - | # | - | - |

| Gene | *Ginkgo biloba* | *Cycas taitungensis* | *Pinus thunbergii* | *Borassus flabellifer* | *Cocos nucifera* | *Elaeis guineensis* | *Hordeum vulgare* | *Oryza sativa* | *Phalaenopsis aphrodite* | *Saccharum officinarum* | *Spirodela polyrhiza* | *Zea mays* | *Arabidopsis thaliana* | *Atropa belladonna* | *Hyoscyamus niger* | *Jatropha curcas* | *Nicotiana tabacum* | *Pisum sativum* |
|---|---|---|---|---|---|---|---|---|---|---|---|---|---|---|---|---|---|---|
| ndhB_398SL | - | - | ^ | + | + | - | - | - | ^ | - | + | - | - | - | - | # | - | - |
| ndhB_419HY | - | - | ^ | - | + | - | - | - | ^ | - | + | - | + | - | - | # | - | - |
| ndhB_494PL | - | - | ^ | + | + | - | + | + | ^ | + | + | + | + | + | + | # | + | - |
| ndhD_129SL | + | - | ^ | + | - | - | # | - | ^ | - | # | - | - | - | - | # | - | - |
| ndhD_326SL | - | - | ^ | + | - | - | # | - | ^ | - | - | - | - | - | - | # | - | - |
| ndhD_399SL | - | - | ^ | + | - | - | # | - | ^ | - | + | - | - | - | - | # | - | - |
| ndhD_438SL | - | - | ^ | + | - | - | # | - | ^ | - | + | - | - | - | - | # | - | - |
| ndhF_21SL | - | - | ^ | - | + | - | # | + | ^ | + | - | + | - | - | - | # | - | - |
| ndhF_97SL | + | + | ^ | - | + | - | # | - | ^ | - | - | - | + | - | - | # | + | + |
| ndhG_116PL | - | - | ^ | + | + | - | # | + | ^ | - | - | - | - | - | - | # | S-L | S-L |
| ndhH_169SL | - | - | ^ | - | + | - | # | - | ^ | - | - | - | - | - | - | # | - | - |
| ndhI_130SF | - | - | ^ | + | - | - | # | - | ^ | - | - | - | - | # | # | # | - | - |
| ndhK_248QST | - | - | ^ | + | - | - | # | - | ^ | - | - | - | - | # | # | # | - | - |
| petB_129AV | - | - | - | + | - | - | # | - | - | - | - | - | - | # | # | # | - | - |
| petB_140RW | - | - | - | + | + | + | # | - | - | - | - | - | - | # | # | # | - | - |
| petB_204PL | - | - | - | + | + | - | # | - | + | + | - | + | - | + | + | # | + | + |
| rpl2_1TM | - | - | - | - | - | + | + | + | + | + | + | + | - | # | # | # | - | - |
| rpl22_83SL | - | - | - | + | - | - | # | - | - | - | - | - | - | # | # | # | - | - |
| rpl23_24SL | - | - | - | - | + | + | # | - | + | - | + | - | - | # | # | # | - | - |
| rpl23_30SF | - | - | - | - | + | + | # | - | - | - | + | - | - | # | # | # | - | - |
| rpoC1_14PL | - | - | - | + | + | - | # | - | - | - | - | - | - | # | # | # | - | - |
| rpoC1_169SL | - | - | - | + | - | - | # | - | - | - | - | - | - | # | # | # | - | - |
| rpoC1_171RW | - | - | - | + | + | - | # | - | - | - | - | - | - | # | # | # | - | - |
| rpoC1_206SL | + | - | - | + | + | - | # | - | - | - | - | - | - | # | # | # | - | - |
| rps3_10FF | - | - | - | + | - | + | # | - | - | - | + | - | - | # | # | # | - | - |
| rps3_157TI | - | - | - | + | + | + | # | - | - | - | - | - | - | # | # | # | - | - |

| Gene | *Ginkgo biloba* | *Cycas taitungensis* | *Pinus thunbergii* | *Borassus flabellifer* | *Cocos nucifera* | *Elaeis guineensis* | *Hordeum vulgare* | *Oryza sativa* | *Phalaenopsis aphrodite* | *Saccharum officinarum* | *Spirodela polyrhiza* | *Zea mays* | *Arabidopsis thaliana* | *Atropa belladonna* | *Hyoscyamus niger* | *Jatropha curcas* | *Nicotiana tabacum* | *Pisum sativum* |
|---|---|---|---|---|---|---|---|---|---|---|---|---|---|---|---|---|---|---|
| rps3_195HY | + | - | - | + | + | + | # | - | - | - | - | - | - | # | # | # | - | - |
| rps7_100AA | - | - | - | + | + | + | # | - | - | - | + | - | - | # | # | # | - | - |
| rps8_61SL | - | - | - | + | + | - | # | + | + | + | + | + | - | # | # | # | - | - |
| ycf3_15SF | - | - | - | + | + | - | - | - | + | - | - | + | ^ | # | # | # | - | - |
| ycf3_62TM | - | - | - | + | + | - | - | + | + | + | + | + | - | # | # | # | - | - |
| ycf3_64PL | + | + | - | + | + | - | - | - | + | - | - | - | - | # | # | # | - | - |
| ycf4_85SL | + | - | - | + | + | + | - | - | - | - | - | - | - | # | # | # | - | - |

+= editing, -= no editing, ^=no gene, #=no report, SL= Serine to Leucine as an alternative editing pattern
