# Supplementary material for: RNA editing in the chloroplast of Asian Palmyra palm (Borassus flabellifer)
Source: Genet Mol Biol. 2020 Jan 13;42(4):e20180371. doi: 10.1590/1678-4685-GMB-2018-0371 (PMC7206934; doi:10.1590/1678-4685-GMB-2018-0371)
Supplement: Supplementary file 3 [file 1415-4757-GMB-42-4-e20180371-suppl4.pdf]

Supplementary Material to: "RNA editing in the chloroplast of Asian Palmyra palm (*Borassus flabellifer*)"

Table S3 - Accessions numbers of cp genomes from 59 plant species**.**

Species	Accession number
Abolboda macrostachya	HQ180528
Alpinia zerumbet	JX088668.1
Baxteria australis	NC_029970.1
Belosynapsis ciliata	HQ180447.1
Bismarckia nobilis	NC_020366.1
Borassus flabellifer	KP901247.1
Brocchinia micrantha	AY614381.1
Calamus caryotoides	NC_020365.1
Calectasia narragara	JX088666.1
Canna indica	KF601570.1
Centrolepis monogyna	EF153936.1
Chamaedorea seifrizii	JX088667.1
Chamaerops humilis	NC_029967.1
Cocos nucifera	KF285453
Costus pulverulentus	KF601573.1
Cyperus alternifolius	HQ180453.1
Dasypogon bromeliifolius	NC_020367.1
Doryanthes palmeri	AY699128.1
Ecdeiocolea monostachys	AY465535.1
Elaeis guineensis	NC_017602.1
Flagellaria indica	AY465537.1
Georgeantha hexandra	HQ180459.1
Hanguana malayana	NC_029962.1
Heliconia collinsiana	NC_020362.1
Joinvillea ascendens	NC_031427.1
Juncus effusus	HQ180463.1
Kingia australis	JX051651.1

Species	Accession number
Maranta leuconeura	KF601571
Mayaca fluviatilis	AF168929.1
Metroxylon warbergii	NC_029959.1
Monocostus uniflorus	KF601572.1
Musa textilis	NC_022926.1
Neoregelia carolinae	HQ180470.1
*Nypa frutican*s	NC_029958.1
Orchidantha fimbriata	KF601569.1
Oryza sativa indica	NC_001776.1
Pharus latifolius	NC_021372.1
Phoenix dactylifera	NC_013991.2
Phytelephas aequatorialis	NC_029957.1
Pigafetta elata	NC_029956.1
Potarophytum riparium	HQ180475.1
Pritchardia thurstonii	NC_029955.1
Puelia olyriformis	NC_023449.1
Puya laxa	AY614388.1
*Ravenala madagascariens*is	NC_022927.1
Ravenea hildebrandtii	HQ180477.1
Sabal domingensis	NC_026444.1
Sparganium eurycarpum	AY465539.1
Streptochaeta angustifolia	EF422978.1
Syngonanthus chrysanthus	HQ180481.1
Thamnochortus insignis	HQ180482.1
Thaumatococcus daniellii	KF601575.1
Thurnia sphaerocephala	HQ180483.1
*Tradescantia ohie*nsis	EF422980.1
Triticum aestivum	KJ592713.1
Typha latifolia	NC_013823.1
*Washingtonia ro*busta	NC_029974.1
Xiphidium caeruleum	JX088669.1
Zingiber spectabile	NC_020363.1
