# Supplementary material for: RNA editing in the chloroplast of Asian Palmyra palm (Borassus flabellifer)
Source: Genet Mol Biol. 2020 Jan 13;42(4):e20180371. doi: 10.1590/1678-4685-GMB-2018-0371 (PMC7206934; doi:10.1590/1678-4685-GMB-2018-0371)
Supplement: Supplementary file 4 [file 1415-4757-GMB-42-4-e20180371-suppl5.pdf]

Supplementary Material to: "RNA editing in the chloroplast of Asian Palmyra palm (*Borassus flabellifer*)"

Table S4. Predicted and experimentally identified RNA editing sites in 35 chloroplast genes of *B. flabellifer*. + = editing site, - = no editing site and # = not predicted by the PREP-Cp.

Gene	Editing position	Edited with in codon position	Base substitution	Edited codon	Amino acid change	Predicted PREP	PREPACT 3.0	Experimental identification
accD_52RW	154	1	c-U	cgg-Ugg	R-W	+	+	-
accD_135HY	403	1	c-U	cat-Uau	H-Y	+	+	-
accD_267SL	800	2	c-U	tcg-uUg	S-L	+	+	-
accD_388SL	1163	2	c-U	tca-uUa	S-L	+	+	-
accD_470PL	1409	2	c-U	cct-cUu	P-L	+	-	+
atpA_365PS	1093	1	c-U	cca-Uca	P-S	+	-	-
atpA_369PS	1105	1	c-U	ccg-Ucg	P-S	+	-	-
atpA_403SL	1208	2	c-U	tcg-uUg	S-L	+	-	-
atpA_424PS	1270	1	c-U	ccc-Ucc	P-S	+	-	-
atpA_429SL	1286	2	c-U	tca- uUa	S-L	+	-	-
atpA_458SL	1373	2	c-U	tcg-uUg	S-L	+	-	-
atpA_472SF	1415	2	c-U	tcc-uUC	S-F	+	-	-
atpB_395SL	1184	2	c-U	tca-uUa	S-L	+	+	+
atpF_31PL	92	2	c-U	cca-cUa	P-L	+	+	+
atpI_143PL	428	2	c-U	ccc-cUc	P-L	+	+	+
atpI_210SL	629	2	c-U	tca-uUa	S-L	+	+	+
clpP_187HY	559	1	c-U	cat-Uau	H-Y	+	+	+
matK_55SF	164	2	c-U	tct-uUu	S-F	+	+	+
matK_63SL	188	2	c-U	tca-uUa	S-L	+	+	+
matK_219PL	656	2	c-U	cca-cUa	P-L	+	-	-
matK_426HY	1276	1	c-U	cat-Uau	H-Y	+	-	+
ndhA_159SL	476	2	c-U	tca-uUa	S-L	+	+	+
ndhA_189SL	566	2	c-U	tca-uUa	S-L	+	+	+
ndhA_358SF	1073	2	c-U	tcc-uUc	S-F	+	+	-
ndhB_50SL	149	2	c-U	tca-uUa	S-L	+	+	+
ndhB_156PL	467	2	c-U	cca-cUa	P-L	+	+	+
ndhB_181TM	542	2	c-U	acg-aUg	T-M	+	+	+
ndhB_196HY	586	1	c-U	cat-Uau	H-Y	+	+	+
ndhB_235SF	704	2	c-U	tcc-uUc	S-F	+	+	+

ndhB_246PL	737	2	c-U	cca-cUa	P-L	+	+	+
ndhB_277SL	830	2	c-U	tca-uUa	S-L	+	+	+
ndhB_279SL	836	2	c-U	tca-uUa	S-L	+	+	+
ndhB_371SL	1112	2	c-U	tca-uUa	S-L	+	+	+
ndhB_398SL	1193	2	c-U	tca-uUa	S-L	+	+	+
ndhB_419HY	1255	1	c-U	cat-Uau	H-Y	+	+	-
ndhB_494PL	1481	2	c-U	cca-cUa	P-L	+	+	+
ndhD_2TM	5	2	c-U	acg-aUg	T-M	+	-	-
ndhD_21SL	62	2	c-U	tca-uUa	S-L	+	+	-
ndhD_129SL	386	2	c-U	tca-uUa	S-L	+	+	+
ndhD_226SL	677	2	c-U	tcg-uUg	S-L	+	+	-
ndhD_687SL	687	2	c-U	tcg-uUg	S-L	-	-	+
ndhD_399SL	1196	2	c-U	tca-uUa	S-L	+	+	+
ndhD_438SL	1313	2	c-U	tca-uUa	S-L	+	+	+
ndhF_21SL	62	2	c-U	tca-uUa	S-L	+	+	-
ndhF_97SL	290	2	c-U	tca-uUa	S-L	+	+	-
ndhF_148HY	442	1	c-U	cat-Uau	H-Y	+	+	-
ndhF_196LF	586	1	c-U	ctt- Uuu	L-F	+	+	-
ndhF_465HY	1393	1	c-U	cac-Uac	H-Y	+	-	-
ndhG_105TI	314	2	c-U	aca-aUa	T-I	+	-	-
ndhG_116PL	347	2	c-U	cca-cUa	P-L	-	-	+
ndhI_130SF	389	2	c-U	tct-uUu	S-F	#	-	+
ndhK_248QST	742	1	c-U	cag-Uag	Q-stop	#	+	+
petB_129AV	386	2	c-U	gca-gUa	A-V	-	-	+
petB_140RW	418	1	c-U	cgg-Ugg	R-W	+	+	+
petB_204PL	611	2	c-U	cca-cUa	P-L	+	+	+
rpl2_1TM	2	2	c-U	acg-aUg	T-M	+	+	-
rpl20_9TI	26	2	c-U	aca-aUa	T-I	+	-	-
rpl20_103SL	308	2	c-U	tca-uUa	S-L	+	+	-
rpl22_83SL	248	2	c-U	tca-uUa	S-L	#	-	+
rpoA_123SL	368	2	c-U	tca-uUa	S-L	+	+	-
rpoA_277SL	830	2	c-U	tca-uUa	S-L	+	+	-
rpoA_296SL	887	2	c-U	tca-uUa	S-L	+	-	-
rpoC1_14PL	41	2	c-U	cca-cUa	P-L	+	+	+

rpoC1_169SL	488	2	c-U	tca-uUa	S-L	-	-	+
rpoC1_171RW	511	1	c-U	cgg-Ugg	R-W	-	+	+
rpoC1_206SL	617	2	c-U	tca-uUa	S-L	+	+	+
rpoC1_406SF	1217	2	c-U	tcc-uUc	S-F	+	-	-
rpoC1_466SF	1397	2	c-U	tcc-uUc	S-F	+	-	-
rpoC1_544SL	1631	2	c-U	tca-uUa	S-L	+	-	-
rpoC1_586PS	1756	1	c-U	cct-Ucu	P-S	+	-	-
rpoC1_616AV	1847	2	c-U	gct-gUu	A-V	+	-	-
rps3_10FF	30	3	c-U	ttc-uuU	F-F	#	-	+
rps3_157TI	470	2	c-U	aca-aUa	T-I	#	+	+
rps3_195HY	583	1	c-U	cat-Uau	H-Y	#	+	+
rps7_100AA	300	3	c-U	gcc-gcU	A-A	#	-	+
rps8_61SL	182	2	c-U	tca-uUa	S-L	+	+	+
ycf3_15SF	44	2	c-U	tct-uUu	S-F	+	+	+
ycf3_62TM	185	2	c-U	acg-aUg	T-M	+	+	+
ycf3_64PL	191	2	c-U	cca-cUa	P-L	+	+	+
ycf4_85SL	254	2	c-U	tca-uUa	S-L	#	+	+
rpl23_24SF	71	2	c-U	ucu-uuU	S-F	#	+	-
rpl23_30SF	89	2	c-U	uca-uUa	S-L	#	+	-
ndhH_169HY	505	1	c-U	cau-Uau	H-Y	#	+	-
ndhH_182SF	545	2	c-U	ucu-uUu	S-F	#	+	-
ycf2_362RW	1084	1	c-U	cgg-Ugg	R-W	#	+	-
psaJ						#	-	-
psbC						#	-	-
psbH						#	-	-
petA						#	-	-
rpl14						#	-	-
rps16						#	-	-
psaB						#	-	-
