# Supplementary material for: RNA editing in the chloroplast of Asian Palmyra palm (Borassus flabellifer)
Source: Genet Mol Biol. 2020 Jan 13;42(4):e20180371. doi: 10.1590/1678-4685-GMB-2018-0371 (PMC7206934; doi:10.1590/1678-4685-GMB-2018-0371)
Supplement: Supplementary file 5 [file 1415-4757-GMB-42-4-e20180371-suppl1.pdf]

Supplementary Material to "RNA editing in the chloroplast of Asian Palmyra palm (*Borassus flabellifer*)"

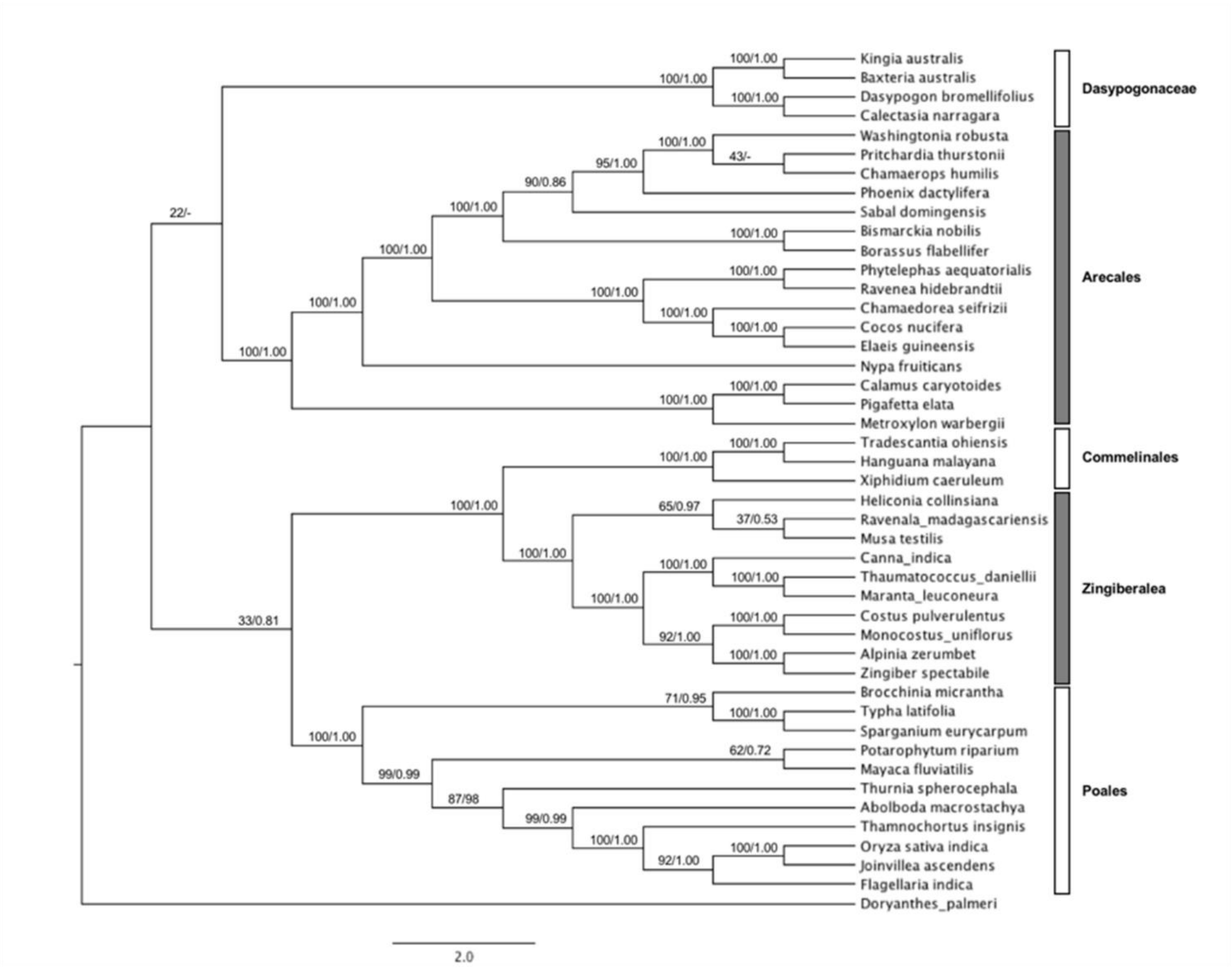

Figure S1 - Cladograms of commelinids reconstructed from 14 cp genes (without ycf1) based on maximum likelihood and Bayesian method with GTR+I+G model.
